# A mixed-method survey to understand the role of dog welfare organisations in Ireland, including reported challenges and potential solutions

**DOI:** 10.1186/s13620-023-00249-6

**Published:** 2023-09-30

**Authors:** Claire McKernan, Catherine Lawler, Blain Murphy, Daniel M. Collins, Simon J. More, Sean Murray, Patricia Reilly, Rob Doyle, Natascha V. Meunier, Aiden Maguire, Locksley L. McV. Messam

**Affiliations:** 1 Institute for Global Food Security, Queen’s University Belfast, Belfast, Northern Ireland; 2 Department of Agriculture, Food and the Marine, Kildare St, Dublin, D02 WK12 Ireland; 3 UCD Centre for Veterinary Epidemiology and Risk Analysis, School of Veterinary Medicine, University College Dublin, Belfield, Dublin, D04 W6F6 Ireland; 4 School of Veterinary Medicine, University College Dublin, Belfield, Dublin, D04 W6F6 Ireland; 5 Animal Health Ireland, 4-5 The Archways, Carrick On Shannon, N41 WN27 Co. Leitrim Ireland

## Abstract

**Background:**

This novel study forms part of a larger research programme seeking an improved understanding of aspects of the owned dog population in Ireland. Dog welfare organisations (DWOs) in Ireland are recognised as an instrumental pillar of the animal welfare sector with some receiving substantial public funding. We conducted a survey of DWOs in Ireland (*n* = 39) to gain a better understanding of their role and function, including their policies and procedures and the rehoming of dogs to other regions. In addition, we wanted to get a better understanding of the challenges experienced by DWOs in fulfilling their role and their perspectives on potential solutions to these challenges. The survey questions consisted of closed and open-ended items. Closed items were analysed quantitively; open-ended items were analysed thematically.

**Results:**

Most DWOs (> 80%) had written protocols for important welfare actions including rehoming procedures, assessment of owner suitability and euthanasia. DWOs sent dogs to Northern Ireland (13%), Great Britain (38.5%) and to other countries outside the United Kingdom (36%, including Germany, Sweden, Italy, the Netherlands and Czechia). Reported challenges included a general lack of funding, limited public awareness of the importance of dog welfare and insufficient capacity to handle dog numbers. To address these challenges, the DWOs highlighted the potential contribution of subsidised programmes and access to resources to educate potential owners. In a further qualitative evaluation to capture perceptions of appropriate solutions by DWOs, several themes emerged, relating to legislation, education, an overwhelmed workforce, and funding.

**Conclusions:**

This study provides important insights into the roles and functions of DWOs and challenges they experience in Ireland. It is hoped that the findings from this research will inform future research investigating potential solutions to these challenges as well as the development of policy in Ireland.

**Supplementary Information:**

The online version contains supplementary material available at 10.1186/s13620-023-00249-6.

## Background

Many dog owners in Europe consider their pet to be a part of the family [1, 2], with 25–35% of Irish households reporting at least one dog in their home [3]. Canine welfare has subjective connotations for different people [4] and its safeguarding involves many interconnected components. Ultimately, maintaining dog welfare includes protecting animals from abuse and neglect, providing sanitary housing, providing an adequately balanced diet and clean water as well as disease control, vaccinations, and access to veterinary care, in addition to regular grooming and exercise [5, 6]. In addition, ensuring psychological and emotional well-being is a fundamental element of animal welfare including addressing an animal's behavioural needs such as exercising natural behaviour, interacting with its own species, and playing [6].

Previous studies on dog welfare have mainly focussed on the owner-dog relationship. Several studies completed in Ireland and Great Britain have highlighted limited awareness and knowledge among dog owners in relation to both guardianship fundamentals (i.e., correct feeding and exercise) [7–9] and the legislation applicable to dog ownership, such as dog identification and tail docking [10]. Anderson et al. reported that legislation in relation to companion animal welfare differed widely across 11 western jurisdictions [11] and studies have suggested that structured education campaigns could contribute to improvements in overall animal welfare. However, the scope of these campaigns are often limited to dog owner attitudes, beliefs and opinions influencing understanding and knowledge [11, 12]. Education while necessary, is not sufficient to ensure behaviour change and translation of knowledge into action. Michie, van Stralen and West describe the COM-B system, which is a behavioural system involving three essential conditions – capability, opportunity, motivation – and nine intervention functions aimed at addressing deficits in one or more of these conditions and interacting to generate behaviour change [13]. Other studies have considered how a dog’s environment, training, and specific exercise (i.e., lead walking) can help to address behavioural issues in dogs, thus contributing to better animal welfare [14–18].

To date, there have been few peer-reviewed studies seeking an understanding of the characteristics of the owned-dog population in Ireland, including overall size and distribution. Downes et al. [19, 20] investigated aspects of dog ownership in Ireland in 2007. At that time, they estimated that 35.6% of Irish households owned one or more pet dogs. Dog ownership was associated with such factors as location, house type, household social class and composition, presence of children in the household, and the presence of a cat [19, 20]. Subsequent published work has mainly focused on Irish legislation and controls [21, 22]. Most recently, Keogh et al. reported that among well-educated employees of an Irish University, there were low levels of awareness (both dog owners and non-dog owners) that key responsibilities of dog owners are stipulated under Irish law [10].

In Ireland, dog welfare organisations (DWOs) receive surrendered dogs from the public, assess dogs for suitability to be re-homed, re-home dogs, and educate potential dog owners. These organisations regularly communicate among themselves and with multiple stakeholders such as local and national government officials and An Garda Síochána (the Irish police and security service). An annual grant is also given to eligible DWOs (those with charity status as determined by the Irish Charities Regulator) by the Department of Agriculture, Food and the Marine (DAFM).

Due to the limited research available in relation to the role and challenges faced by these organisations, and their reliance on public funding, DWOs were chosen as the focus of this study. The current study is part of a multi-study research programme seeking to develop a robust evidence base in relation to the owned dog population in Ireland, including challenges and opportunities for dog ownership and welfare, and the role played by DWOs, noting that some may also care for other animal species. As part of this research programme, More et al. reviewed the usefulness of existing data sources to inform our understanding of changes to the pet dog population in Ireland, including those relating to biological (demographics, movement of dogs across national borders) and organisational (the roles of different organisations, regulatory and non-regulatory impacts, drivers of supply and demand) processes [23]. Further, Murphy et al. identified and explored the experiences of DWOs in Ireland using a qualitative study design integrating online focus groups and interviews [24]. In the current study, a mixed-methods approach was used to seek additional information in relation to DWOs in receipt of animal welfare grant funding in Ireland. In particular, the current study sought to describe the roles and functions of selected DWOs in Ireland using quantitative items, such as general information about the organisations, dogs under their care, and policies and procedures, coupled with qualitative free text options to explore the challenges faced by these organisations and the suitability of solutions available.

## Methods

### Survey design

A survey was developed to capture and gain a better understanding of the role and function of DWOs in Ireland, including their policies and procedures and the rehoming of dogs to other regions, in addition to the challenges they experienced in fulfilling their role and potential solutions to these challenges. The scope and focus of the survey were informed by a narrative review of relevant literature [19], and detailed discussions within the research team. The finalised survey was developed using Qualtrics software (Qualtrics XM, USA), which is available upon request.

The survey was separated into four different sections including:

- General information about the DWOs,
- Information about the DWO’s current policies and procedures,
- Information about the rehoming of dogs to other regions (Northern Ireland (NI), Great Britain (GB), and other countries) in 2019 and 2021, and
- The views of DWOs on the challenges they experience in seeking to positively impact the welfare of dogs, and of potential solutions to address these challenges. This includes the duty of owners to protect animal welfare, the prohibition of animal abandonment and cruelty, the regulation of particular surgical procedures, as well as the requirement of dog licensing, the prohibition of dog straying and dog by-laws, as reported by Keogh et al. [10]

The survey included both closed and open-ended items. Data were collected using 5-point Likert scale items, rank scales, dichotomous “yes” or “no” items, and multiple-choice questions with fixed-choice response options (multiple answers possible). Additionally, fixed-choice response options provided an “Other, (please specify)” item to obtain further qualitative data. Free-text items were included to capture qualitative insights from DWOs to support findings from the quantitative analysis and to provide further insights on proposed solutions to challenges they experience. The survey questions are included as Supplementary material.

A pilot survey was conducted with individuals working within the dog welfare environment, to ensure items were understood as intended by the research team and conducive to reflecting participants’ experiences. Following this pilot survey, the number of items in the survey was substantially reduced to alleviate participant burden.

### Data collection

The target population included the 68 DWOs that received animal welfare grant funding from the DAFM in 2021. One representative per organisation was invited to participate in the study, which was conducted during March–April 2022. An initial email was circulated to a generic in-box for each of these DWOs to introduce and outline the purposes of the study. Subsequent to this initial contact, a representative from each of the responding organisations volunteered to take part in the study on behalf of the organisation that they were affiliated with. In addition, weekly follow-up emails were sent for 3 successive weeks to encourage participation. To maximise participation, the survey was made available on three different platforms including a self-administered online version, an interviewer-administered version via telephone and a hard copy version (mailed to participants upon request).

With the online version, data were collected directly using the Qualtrics software. If participants opted to complete the survey via telephone or hard-copy questionnaire, their responses were manually inputted into Qualtrics by the first author (CMcK) and labelled accordingly. At the commencement of the survey, each participant was given an explanation of the purpose of the survey. Each participant was also given assurances that their answers would be treated confidentially, and their organisation would not be individually identified in any research. Exemption from ethical review was granted by University College Dublin (UCD) Human Research Ethics Committee (LS-E-21–279-More). Data collection was conducted in accordance with the General Data Protection Regulation (GDPR) guidelines given in the Declaration of Helsinki.

### Data analysis

The raw survey data were exported from Qualtrics into IBM SPSS Statistics version 26.0 (IBM Corporation, Armonk, NY, USA) for analysis. The imported datasets were cleaned prior to analysis, and responses considered partially completed and/or duplicated were removed. Survey responses that did not progress beyond the consent items within 3 weeks of becoming active were recorded as 'blanks' and removed as no organisational information was obtained. Descriptive statistics (frequency, percentages) were used to examine the data with confidence intervals obtained using exact methods and the method by Sison and Glaz for binomial and multinomial proportions, respectively [25, 26]. Free text survey items were imported to NVivo (QSR International Pty Ltd, Doncaster, Victoria, Australia) and data were analysed qualitatively using inductive thematic analysis [27].

The funding awarded to the 68 DWOs in 2021 was retrieved from publicly available information published annually by DAFM. The Odds ratio (OR) for the association between funding level and participation by these DWOs was estimated in order to investigate the potential for systematic differences in DAFM funding levels among those DWOs that did and did not participate in the survey. During this analysis, the DWOs were categorised by survey participation (organisations with completed and partially completed survey responses and organisations with no participation) and 2021 funding level (≤ €20,000, > €20,000).

## Results

The survey was circulated to all 68 eligible organisations. Two of these responses were completed via postal method, and the remaining participants completed the survey online. Initial cleaning removed blank responses (*n* = 13) and duplication (*n* = 11) from the database. Additionally, the responses of five DWOs who partially completed the survey were not included as the proportion of the survey completed was between 2 and 24%, indicating that the participant did not progress beyond providing general organisation information such as name and location. Therefore, 39 DWOs providing complete responses, equating to a 57% response rate.

With respect to all 68 DWOs, there was a slight negative association between participation and 2021 funding level (OR = 0.9; 95% CI: 0.3, 2.4). The estimate is imprecise and the confidence interval is compatible with both a positive and negative association between DAFM funding and study participation in almost equal measure. On average, the survey took 60 min.

The 39 DWOs providing complete responses were located in 19 of the 26 counties in Ireland: Munster (16), Leinster (15), Connacht (5) and Ulster (3).

Only these 39 DWOs were considered further.

### General information on organisation structure, policies and procedures

Of the 39 DWOs with complete responses, most also cared for cats (90%; 95% CI: 76, 97%) and poultry/other birds (31%; 95% CI: 17, 48%). Most DWOs cared for more than one species, with (*n* = 12) (31%; 95% CI: 17, 48%) caring for 2 species, and (*n* = 23) (59%; 95% CI: 42, 74%) caring for 3 or more species. The most common wildlife species (*n* = 16) cared for, included foxes (*n* = 9), hedgehogs (*n* = 5), deer, badgers, otters and bats (*n* = 2).

Few (13%; 95% CI: 4, 24%) of the 39 DWOs were members of the Association of Dogs and Cats Homes (ADCH) and most (74%; 95% CI: 58, 87%) reported using foster homes to provide care for dogs in 2021. A high proportion of DWOs had written protocols for welfare actions, including rehoming procedures (97%; 95% CI: 86, 100%), assessment of owner suitability (92%; 95% CI: 79, 98%), and euthanasia (85%; 95% CI: 69, 94%). In relation to general protocols for animals under their care, approximately half of the DWOs had written procedures for feeding routines, housing, and cleaning (Table 1). DWOs were asked to report the most common reasons for euthanasia in 2019 and 2021 respectively. Dog bites and aggression were reported as the most common reason for euthanasia (n = 20), closely followed by physical injury (*n* = 18), while being unable to rehome dogs was the least common reason and only selected by two organisations. In cases in which DWOs selected 'Other' as their most common reason for euthanasia, the reasons included illness, old age, and no quality of life.

**Table 1:** The number and percentage (with 95% confidence limits (CLs)) of 39 dog welfare organisations with written protocols for welfare actions and general caring procedures. These data relate to organisations in Ireland with complete responses to a survey conducted in March–April 2022

		**Number**	**Percentage**		
	**Item**			**95% CL**	
				**Lower**	**Upper**
Welfare Actions	Euthanasia	33	84.6	69.5	94.1
	Rehoming	38	97.4	86.5	99.9
	Assessment of potential owner suitability	36	92.3	79.1	98.4
	Record of all dogs adopted	38	97.4	85.6	99.9
	Follow up rehoming check	33	84.6	69.5	94.1
General Caring Procedures	Feeding Routines	19	48.7	32.4	65.2
	Housing	22	56.4	39.6	72.2
	Cleaning	23	59.0	42.1	74.4

### Rehoming procedures

At the time of the survey, in 2022, 38 of 39 of the DWOs rehomed dogs. At this time, each of these DWOs followed one or more specific procedures as outlined in Table 2. The procedures most commonly reported by these DWOs included a home visit (100%; 95% CI: 91, 100%), a check on existing animals residing in owners’ homes (95%; 95% CI: 82, 95%) and verification of owner experience with companion animals (79%; 95% CI: 63, 90%). Most of these organisations employed multiple procedures before releasing a dog to a new owner, with 11 DWOs including three procedures (29%; 95% CI: 15, 46%) and 17 DWOs including four or more (45%, 95% CI: 29, 62%). Participants that selected ‘Other’ in response to the question about releasing a dog to a new owner, indicated that home visits were not permitted due to the recent COVID-19 pandemic. Four other DWOs implemented an online form or questionnaire for dog adoption to assess owner suitability, and one DWO sought references from the private veterinary practitioner. Interestingly, one DWO indicated that they would like to have the capability to determine if a potential owner has convictions in relation to animal cruelty (however, this is not currently possible under the GDPR).

**Table 2:** The number and percentage (with 95% confidence limits (CLs)) of 38 dog welfare organisations that conducted procedures when a dog was released to a new owner. These data relate to organisations in Ireland with complete responses to a survey conducted in March–April 2022 that re-homed dogs

	**Number**	**Percentage**		
			**CL (95%)**	
**Procedures conducted**			**Lower**	**Upper**
Home visit	38	100.0	90.7	100.0
Check (or verification) of the number and type of animals already in the home	36	94.7	82.2	94.7
Verification of prospective owner experience with dogs or other companion animals	30	78.9	62.7	90.4
Organisation of trial visits (with prospective owners)	24	63.2	46.0	78.2
Screening of prospective owner for previous breaches of animal welfare legislation	7	18.4	7.7	34.3
Request for proof of income to determine ability to provide proper care	2	5.3	0.6	17.3

In relation to fees for rehoming dogs, the majority of the 38 DWOs (64%) requested a fixed amount, and some requested a voluntary donation (26%). The two participants that selected “Other”, indicated that finding a suitable owner and home for a dog is much more important than the financial situation of the owner. One DWO indicated that the fixed amount requested, assisted with necessary neutering, microchipping, and vaccination costs. Additionally, most DWOs reported having a written record of all dogs adopted (97%; 95% CI: 86, 100%) and completed a follow up rehoming check (85%; 95% CI: 70, 94%) (Table 1). Most DWOs (84%) indicated that social media (Facebook, Instagram) were the most effective and influential platforms to use for rehoming dogs.

### Rehoming dogs to other regions

Most of the 39 DWOs reported that they did not send dogs to other countries during either 2019 or 2021. Dogs were reportedly sent by DWOs to GB (England, Scotland, and Wales) (39%; 95% CI: 23, 55%), other countries outside the UK (36%; 95% CI: 21, 53%), and NI (13%; 95% CI: 4, 27%) (Table 3).

**Table 3:** The number and percentage (with 95% confidence limits (CLs)) of 39 dog welfare organisations that sent dogs to selected countries (2019 and 2021). These data relate to organisations in Ireland with complete responses to a survey conducted in March–April 2022

Country	Number	Percentage		
			**95% CL**	
			**Lower**	**Upper**
Great Britain	15	38.5	23.3	55.4
Northern Ireland	5	12.8	4.3	27.4
Other^a^	14	35.9	21.2	52.8

^a^ Includes Germany, Sweden, Italy, the Netherlands and Czechia

Participants were further asked to indicate the three countries, excluding GB and NI, to which dogs were most frequently rehomed. For the purposes of this study, the data for 2019 and 2021 were consolidated when identifying countries and determining the frequency of movements. Among the 14 DWOs that rehomed dogs to countries other than the UK during 2019 and 2021, the most common destinations were Germany (31%, *n* = 4), Sweden (31%, *n* = 4), Italy (23%, *n* = 3), The Netherlands (7%, *n* = 1) and Czechia (7%, *n* = 1) (Table 3).

The most common reason for rehoming dogs to NI was the perception that there are more suitable owners in NI compared to the Republic of Ireland. Overall DWOs that sent dogs to GB and other countries did so because of a perception of insufficient eligible owners in Ireland (Table 4). Among participants that selected “Other”, one reported that “*Irish farmers keep breeding to get a good sheepdog. No one wants collies in Ireland; in the UK, they love them and there is more of a demand*”. Another participant indicated that they “… *only send dogs over to UK or any other country if an owner comes forward from that country. We do not seek new owners from other countries for the simple reason that other countries cannot be the solution for the Irish dog problem*”.

**Table 4:** The number and percentage of reasons reported by dog welfare organisations for rehoming dogs outside Ireland, for 15 organisations that rehomed to Great Britain (GB), 5 to Northern Ireland (NI) and 14 to other countries (Germany, Sweden, Italy, the Netherlands and Czechia). These data relate to organisations in Ireland with complete responses to a survey conducted in March–April 2022 that rehomed to these countries

Reasons	N (%)		
	**GB (*n* = 15)**	**NI (*n* = 5)**	**Other countries (*n* = 14)**
Insufficient eligible owners for rehoming dogs in Ireland	10 (66.7)	2 (40.0)	10 (71.4)
More suitable owners for rehoming dogs in NI/GB/Other countries	10 (66.7)	3 (60.0)	7 (50.0)
More suitable charities/organisations in NI/GB/Other countries	10 (66.7)	2 (40.0)	7 (50.0)
Financial contributions for rehoming of dogs higher in NI/ GB/Other countries	0 (0.0)	0 (0.0)	0 (0.0)
Contractual agreements with funders or other agencies/charities	0 (0.0)	0 (0.0)	0 (0.0)
Other	2 (13.3)	2 (40.0)	2 (14.3)

### Challenges experienced, and solutions suggested, by dog welfare organisations

In total, 32 (82%; 95% CI: 66, 92%) DWOs either agreed or strongly agreed that all animal welfare organisations should be registered with the Charities Regulatory Authority. Similarly, most DWOs (82%; 95% CI: 66, 92%) agreed or strongly agreed that minimum operational and animal welfare standards should be established by the regulatory authority (DAFM).

When the 39 DWOs were asked to state the extent to which they experienced selected challenges in fulfilling their roles and positively impacting dog welfare on a Likert scale (from strongly agree to strongly disagree), 82% (95% CI: 72, 93%) either agreed or strongly agreed with the assertion that funding was insufficient (Table 5). Moreover, 31 (80%; 95% CI: 69, 92%) either agreed or strongly agreed that limited public awareness in relation to the importance of dog welfare presented a challenge, 30 (77%; 95% CI: 67, 91%) agreed or strongly agreed that there was insufficient capacity to handle the number of dogs, and 25 (64%; 95% CI: 51, 80%) agreed or strongly agreed that insufficient staffing levels posed a problem (Table 5). Participants that selected “Other” to this question, reported that *"difficulties in persuading dog owners to comply with legislation/rules"* and *"government bodies not enforcing regulations (e.g., microchipping and dog licensing)"* were significant challenges. Twenty percent or more of the DWOs disagreed or strongly disagreed that insufficiently trained staff, insufficient coordination with other DWOs, insufficient engagement with local authorities and difficulty with complying with government requirements were challenges they faced (Table 5).

**Table 5:** Percentages (%) of 39 dog welfare organisations that Agree or Strongly Agree, Neither Agree nor Disagree, and Disagree or Strongly Disagree that they experience selected challenges in fulfilling their roles

Challenges	Response	n (%)
General lack of funding	Agree or Strongly Agree	32 (82)
	Neither Agree nor Disagree	3 (8)
	Disagree or Strongly Disagree	4 (10)
General lack of awareness among the public of the importance of dog welfare	Agree or Strongly Agree	31 (80)
	Neither Agree nor Disagree	4 (10)
	Disagree or Strongly Disagree	4 (10)
Insufficient capacity to handle the number of dogs (supply and/or demand)	Agree or Strongly Agree	30 (77)
	Neither Agree nor Disagree	6 (15)
	Disagree or Strongly Disagree	3 (8)
Difficulties or an inability to rehome particular dog breeds	Agree or Strongly Agree	32 (82)
	Neither Agree nor Disagree	4 (10)
	Disagree or Strongly Disagree	3 (8)
Insufficient staff	Agree or Strongly Agree	25 (64)
	Neither Agree nor Disagree	8 (21)
	Disagree or Strongly Disagree	6 (15)
Lack of resources to meet costs of rehoming abroad (certification, transport, etc.)	Agree or Strongly Agree	21 (54)
	Neither Agree nor Disagree	13 (33)
	Disagree or Strongly Disagree	5 (13)
Difficulties for organisation to comply with government requirements	Agree or Strongly Agree	21 (54)
	Neither Agree nor Disagree	6 (15)
	Disagree or Strongly Disagree	12 (31)
Insufficient engagement with local authorities	Agree or Strongly Agree	20 (51)
	Neither Agree nor Disagree	8 (21)
	Disagree or Strongly Disagree	11 (28)
Lack of sufficient coordination with other DWOs	Agree or Strongly Agree	18 (46)
	Neither Agree nor Disagree	10 (26)
	Disagree or Strongly Disagree	11 (28)
Insufficient staff with suitable training	Agree or Strongly Agree	16 (41)
	Neither Agree nor Disagree	15 (39)
	Disagree or Strongly Disagree	8 (20)

When the 39 DWOs were asked to indicate their opinion on specific solutions to address challenges experienced, almost all (95%; 95% CI: 90, 100%) indicated that subsidised programmes involving vaccination, neutering, and microchipping would be very or extremely helpful (Table 6). Further, 30 (77%; 95% CI: 67, 91%) felt greater clarity about the criteria used when awarding government grant funding would be very helpful or extremely helpful (Table 6). Solutions promoting increased education on animal welfare were also perceived to be beneficial by the DWOs with 80% (95% CI: 69, 92%) indicating that access to standardised training for volunteers/employees and 82% (95% CI: 72, 93%) indicating that access to resources to educate owners on breed suitability would be helpful or extremely helpful. In addition, 30 (77%; 95% CI: 67, 91%) organisations felt that rigorous enforcement of recommendations and policies would be very helpful or extremely helpful (Table 6). Overall, subsidisation of programmes (e.g., vaccination, neutering and microchipping) were felt to be potentially the most helpful solutions with no DWO feeling this would not be so (Table 6).

**Table 6:** Percentages (%) of 39 dog welfare organisations that feel that selected solutions would be helpful in addressing challenges they experience in fulfilling their roles

Potential Solutions	Response	n (%)
Subsidised programmes (vaccination, neutering & microchipping)	Very or Extremely Helpful	37 (95)
	Slightly or Moderately Helpful	2 (5)
	Not at all Helpful	0 (0)
Access to resources to educate potential owners (i.e., breed suitability)	Very or Extremely Helpful	32 (82)
	Slightly or Moderately Helpful	7 (8)
	Not at all Helpful	0 (0)
Rigorous enforcement of recommendations/policies	Very or Extremely Helpful	30 (77)
	Slightly or Moderately Helpful	7 (8)
	Not at all Helpful	2 (5)
Access to standardised training for volunteers and employees	Very or Extremely Helpful	31 (80)
	Slightly or Moderately Helpful	2 (5)
	Not at all Helpful	6 (15)
Greater clarity on the criteria for government financial grants	Very or Extremely Helpful	30 (77)
	Slightly or Moderately Helpful	8 (20)
	Not at all Helpful	1 (3)
Opportunity to attend conferences or seminars with other welfare organisations	Very or Extremely Helpful	27 (69)
	Slightly or Moderately Helpful	11 (28)
	Not at all Helpful	1 (3)

In response to an open-ended question aimed at capturing perceptions of appropriate solutions specifically related to challenges experiences by DWOs to fulfil their role, several themes emerged, relating to legislation, education, an overwhelmed workforce, and funding. These themes each reflect the opinions and beliefs of the DWOs.

### Legislation

The majority of DWOs believed that there was limited compliance with legislation among dog owners. Moreover, they felt that legislation, such as that relating to microchipping, is not uniformly or stringently enforced.

“*Modify the legislation to allow for on the spot fines for non-compliance, for example, a €100 fine for not* <*having*> *a dog microchipped”*

*“Anyone adopting a dog from any charity should produce a current dog licence when collecting their new dog, to bring regulation to dog ownership. We know how many cattle we have in Ireland but when it comes to dogs, it is guesswork. The micro chipping registrations need to be seriously looked at; it is the responsibility of the new owner to re-register the dog in their name”*

The DWOs raised concerns about a perceived lack of awareness amongst the Gardaí (Irish police) regarding the regulations outlined by the Animal Health and Welfare Act 2013 (Act 15/2013), and sought greater collaboration and engagement with local authorities, including local dog wardens*.*

*“While Gardaí are authorised officers under the 2013 Animal Welfare Act, most are unaware of this and regularly pass what are actually crimes under this Act, to animal welfare societies who lack the power to do anything about them”*

*“More involvement and engagement with local authorities and Garda with animal rescue groups in terms of assisting with difficult dog cases, cases of suspected cruelty and neglect”*

The DWOs cited over-breeding and unregulated puppy-farming for fashionable dog breeds as a serious challenge and called for the introduction of stricter regulations on breeding.

*“By far the biggest issue facing animal welfare in Ireland is over-breeding. In the case of dogs, whether that be the breeds typically associated with conventional puppy farming e.g., 'Cockerpoos', Maltipoos etc which are nothing more than mongrels at the end of the day, along with puppy-farmed toy breeds, factor in the strain the Greyhound industry with its culture of overbreeding this causes considerable stress for rescues….Because Ireland has such an established reputation as the puppy farming capital of Europe, there is a strong movement of young breeding dogs brought into Ireland for 'Backyard Breeding' too. So a significant/total shut down of commercial puppy farming, including Greyhounds is needed”*

### Awareness and education

The DWOs highlighted a perceived lack of awareness amongst the general public regarding the importance of dog welfare and the responsibilities of dog ownership. These organisations believed that there was a need to educate the general public about animal welfare and dog breed suitability and highlighted a need for animal welfare elements to be added to the school curriculum at primary and secondary level.

*“More education is needed for the general public in terms of animal welfare, and ability to care and manage dogs, especially those dogs that are on the restricted dog breed list.”*

In addition to public education campaigns, the DWOs recognised the importance of standardised training for employees and volunteers and acknowledged that training personnel is a labour-intensive task for an already stretched workforce.

*“Access to resources and training for those who are involved in animal rescue especially when handling nervous, aggressive, fearful dogs, and injured dogs, and ability to assess behaviour and temperament of dogs.”*

### Overwhelmed workforce

The DWOs reported feeling overwhelmed with their workload and struggled to keep up with the many moving components required. Participants felt that their organisational structure is reliant on volunteers and that time spent completing paperwork frustrated dedicated individuals.

*“The amount of paperwork, forms and unnecessary form filling achieves nothing for me and is seriously crippling. I am putting in a 14 hour day, 7 days a week with no let up.*”

*“I know from personal experience of running the rescue, and indeed other rescues are the same. It’s all falling on few people, who at this stage are burnt-out.”*

Several of the DWOs believed that standards should be harmonised for rescue organisations, to safeguard dog welfare, and that a shared hub or centralised base would be beneficial to encourage collaboration and communication in order to maximise resources.

*“There are no standards set for rescues, everyone needs to be coming from the one place.”*

*“To go forward each welfare organisation needs a Centre/base of its own. Maybe a centralising of one good centre per county would work but welfare people are not good at working together. There is a lot of duplication and also missed opportunities. For example, one organisation has a specific dog, and another has a home for such a dog or breed, there is a huge lack of communication between organisations. Sharing …expertise in behavioural issues. We need a central hub that we can all feed into to share knowledge and to get help. Ireland is small and I believe we could do a much better job if a lot of things were centralised.”*

### Funding

The DWOs reported financial strain, including those associated with veterinary bills and having a consistent team of volunteers. Participants believed that financially subsidised vaccination programmes and a financially subsidised programme for staff/volunteers would assist to encourage sustained involvement and to alleviate workforce burden.

“*Subsidised vaccination, neutering and microchipping for animal welfare organisations would take a lot of stress away. Less fundraising to do, and more time to deal directly with animal welfare cases”*

*“The biggest challenge we face is getting volunteers to help out … if the government offered incentives for people to volunteer to help registered charities, we feel that we would be more effective in the work we do as we would have more available people*.”

## Discussion

To the authors’ knowledge, this study is the first to examine, and gain insights into, the roles and functions of DWOs in Ireland, in addition to obtaining a greater understanding of current challenges experienced by them as well as their views of the suitability of proposed solutions. The results indicated that most organisations care for more than one species. Most DWOs implemented multiple procedures to rehome dogs and conducted follow-up checks, in addition to having written animal welfare protocols. This demonstrates the willingness and engagement of DWOs with procedures to ensure good dog welfare. There was a widespread view among the DWOs that the main challenges experienced in relation to their work in Ireland related to funding, poorly enforced legislation, limited public knowledge and awareness, and an overwhelmed workforce. Therefore, it is unsurprising that, when participants were asked what would help to make changes, financially subsidised programmes, and access to resources to educate potential owners were considered most helpful.

This study provides insights into good welfare practices amongst DWOs, with most organisations reporting the use of written records in relation to euthanasia, rehoming, adoption, and assessment of potential owner suitability. Half of the DWOs had a written protocol for standard operating procedures such as feeding routines, housing, and cleaning. In the current study, the most popular rehoming procedure included pre-adoption home visits, which is in agreement with other studies [28]. DWOs believed that standardising minimum operational and animal welfare practices for all animal rescue organisations would be beneficial, as current practices vary widely. This is consistent with findings in other studies reporting that rehoming organisations regularly engage in some form of screening process to assess dog suitability but to a lesser extent, the screening of eligible adopters [28, 29]. Moreover, some studies reported concerns in relation to the quality of the rehoming assessment processes, coupled with the variability in rehoming procedures [28]. Standardisation of guidelines and recommendations informed by existing guidelines created by organisations such as the ADCH could encourage a consistent approach to promote dog welfare in Ireland. Membership of ADCH, which aims to “safeguard animal welfare”, requires the provision of minimum standards and standard operational procedures, including care of animals, assessment of animals, and animal departures such as rehoming, fostering and euthanasia (adch.org.uk) [30]. The results of this study also indicate that some charities sent dogs to NI, GB, and other jurisdictions, such as Sweden, Germany, Italy, the Netherlands and Czechia, similar to findings reported by More et al. [23]. It is evident that there is a lack of consistency in the approach used by DWOs to assess dog behaviour and potential suitability for rehoming. While guidelines and criteria outlined by ADCH are sound, due to their high-level nature, their application and implementation by DWOs is likely to be variable.

Previous studies suggest that the general public both locally and abroad (e.g., United Kingdom, Denmark and the United States of America) have limited knowledge and awareness of the responsibilities associated with dog ownership [1, 29, 31]. Further, within European countries such as Spain, Czechia and the UK, there are differing standards and attitudes towards dogs, suggesting that both scientific evidence and cultural considerations may be necessary in order to improve the welfare of companion animals [32]. Similarly, in the current study, 80% of DWOs believed that the general lack of awareness of the importance of dog welfare was a substantial challenge. Earlier studies have identified increasing knowledge and awareness as an effective approach to prompting behaviour change with regard to human welfare-related behaviours such as smoking cessation, healthy eating and, more recently, antimicrobial use in agriculture. These studies also highlighted the importance of identification of suitable platforms and tailored educational campaigns as fundamentally important for engagement [33–36]. Therefore, it follows that educational campaigns are a logical approach to increase knowledge among the general public in relation to animal welfare. Studies have assessed the impact of such programmes in a school setting, reporting that involvement in educational programmes on companion animals, wildlife and farm animals increased knowledge in relation to animal welfare, demonstrating the potential for inclusion of such interventions in the school curriculum [37, 38]. Theoretical frameworks such as the COM-B model recognise that education alone is not enough to encourage efficient behaviour change [13], therefore future research should focus on the utilisation of theoretical frameworks to successfully design interventions to achieve this [13].

In the current study, 82% of DWOs believed that access to resources to educate owners on breed suitability would be valuable. This agrees with a recent report highlighting poor matching of breed to owner lifestyle as a problem [39]**.** However, further research is needed to determine the effectiveness of this type of education in changing human behaviour [13]. The Blue Cross has made information available online to help individuals choose the dog breed appropriate for their circumstances [40]. Similarly, the Peoples Dispensary for Sick Animals (PDSA) provides a short survey to assess the type of pet suitable to an individual's lifestyle. This platform also has an interactive page where individuals can look at specific elements such as “basic training for puppies” and “canine body language” (Saving pets, Changing lives—PDSA) [41]. Although it is reassuring that these named organisations provide information to the general public, the type, amount, and techniques for delivering knowledge and advice on these platforms varies widely. While results for the current study called for a greater need to increase dog suitability awareness, some DWOs believed that a centralised hub to share information and resources could be an opportunity for dog welfare charities to develop an interactive platform to allow potential dog owners to access reliable and relevant information and advice on regulation, behaviour and breed suitability.

Volunteers play a key role in DWOs in Ireland, with many organisations mainly reliant on voluntary involvement. A variety of reasons motivate volunteers to become involved with charities, including a sense of purpose, increased confidence, self-esteem, and particular skill sets, in addition to serving as a social network with other people passionate about making a positive contribution to a charity's mission [42, 43]. Studies in Australia have acknowledged that the feeling of burnout is frequently reported in volunteers in the form of perceived low accomplishment and emotional and physical exhaustion [44, 45]. In the current study, DWOs reported feeling overwhelmed by the workload and the volume of paperwork associated with being involved with dog welfare, with organisations suggesting that an incentive programme to increase the volunteer workforce would significantly ease workload and reduce burnout amongst affiliated personnel. As previously discussed, DWOs called for standardising minimum operating procedures. In all likelihood however, this would contribute to more paperwork which, as participants have expressed, is already overwhelming. This apparent contradiction will require further inquiry to ascertain which elements of paperwork are deemed ‘unnecessary’ and which elements could be refined and reviewed in the future. Furthermore, as previously mentioned, a centralised hub to encourage collaboration between DWOs could be valuable to provide a supportive network where organisations may share experience and knowledge.

Results of the current study identified aggressive dog behaviour as the most frequent reason for euthanasia in DWOs. Several studies conducted in NI also reported that dogs exhibiting undesirable behaviour were significantly more likely to be surrendered to dog rescue organisations and were more likely to have longer stays in welfare facilities [46, 47]. While the study by Rooney et al. focused on working dogs rather than rescue dogs, the adoption of evidence-based behaviour modification approaches by properly accredited pet behavioural counsellors is likely to benefit rescue dogs as well [48]. Participants in the current study felt that there was a need for standardised training for dog welfare personnel (staff and volunteers), specifically in relation to handling dogs exhibiting undesirable behaviours. There is the possibility that accredited and specialised organisations may be able to assist in this regard, through training and support of the workforce of DWOs. Wells and Hepper supported this concept and reported that dogs rescued from shelters are generally more likely to display behavioural problems leading to their return to DWOs and acknowledged that raising public awareness of the value of behavioural therapy schemes would improve the situation and assist the transition of dogs to new homes [47]. We note that this approach may not be straightforward given the difficulties in reliably identifying individuals to do the training. Currently, there is no publicly available register of relevant accredited professionals in Ireland.

Previous studies in Ireland and Italy reported that dog owners exhibit limited awareness of their responsibilities to comply with relevant dog welfare legislation, specifically in relation to microchipping and licencing [10, 19, 20, 49]. This is consistent with the findings of the current study, with over 80% of DWOs suggesting that more rigorous enforcement of legislation would be helpful. DWOs reported a lack of awareness among Gardaí in relation to animal welfare issues under the Animal Health and Welfare Act 2013. This perhaps suggests a misperception or lack of knowledge about the precise remit of the Gardai as far as animal welfare is concerned, and a disconnect or communication gap between DWOs and Gardaí. Based on qualitative results from the current study, some DWOs would like increased engagement with local authorities and authorised animal welfare officers in relation to dog cruelty cases. Existing fragmentation of microchip databases, coupled with the absence of a universal database for dog microchipping, has been identified as a major obstacle to accurately ascertaining the size of the dog population in Ireland and the UK [23, 50]. More et al. note that a national database, including dog licence and microchip information, could contribute to increased compliance and assist relevant authorities such as the Gardaí and dog control officers in enforcing legislation [23]. In addition, efforts should be made to address the perceived disconnect between DWOs and authorised officers. For instance, coordination of information between the DWOs, Gardaí, dog wardens and DAFM would be valuable to foster partnerships and strengthen a unified understanding of which actions need to be taken with animal welfare cases.

The DWOs are seeking increased funding to alleviate costs associated with animal welfare expenses such as veterinary bills. Similarly, they highlight the importance of subsidised funding to promote neutering, microchipping and vaccination, as potential solutions to existing challenges. It is important to note that this request is being made in the context of substantial existing government support, noting that each participating DWO currently receives funding through DAFM animal welfare grants, which are utilised to subsidise neutering microchipping and vaccinations. We accept that vaccination programmes are an effective intervention, long established in the animal health care system and recently championed in agriculture, with positive aims including reduced veterinary fees and reduced disease occurrence [36, 51]. The focus of these studies was on commercially managed animals, so further research is needed to determine if the findings are applicable to companion animals.

To our knowledge, this is the first study to utilise a mixed method survey design to investigate roles and functions of DAFM-funded DWOs, and to obtain insights into their challenges and perceptions of potential solutions to these challenges. Given that the reference population included all 68 DWOs that received funding from DAFM in 2021, we accept that participating DWOs may not be representative of those DWOs that did not receive DAFM animal welfare grants. Further, the sample size was small, with a response rate of 57%, which affects the precision of our estimates and may be subject to selection bias [52]. Nevertheless, the weak association between study participation and 2021 funding level, provides confidence that participation was not unduly influenced by the level of funding from DAFM, which sponsored this study. A recent study reported that low response rates in veterinarians may be due to high workloads, which hinder participation in research [52]. It is possible that this could also have been the case for the DWO representatives  in this instance. This study provides an initial and important insight and understanding of the roles and functions of DWOs in Ireland, the challenges they face in fulfilling their roles, and their perceptions of potential solutions. This is an understudied field of inquiry and provides direction for future research. The outputs from this study should help to guide future research in the Irish dog welfare landscape in Ireland and to inform the development of policies to address challenges highlighted in this study.

## Conclusion

In conclusion, most DWOs self-report the implementation of good practices and a dedication to the safeguarding of dog welfare in Ireland. Overall, the dominant challenges reported by them are linked to perceptions that legislation is poorly enforced, that there is limited awareness and knowledge among dog owners about dog welfare, in addition to concerns about non-uniform organisational procedures, financial constraints and an overwhelmed workforce. In response to these challenges, DWOs report that careful consideration of tailored educational campaigns, both for the general public and DWOs, may help to alleviate these challenges. Moreover, fostering relationships between organisations and other relevant local authorities such as dog wardens and the Gardaí could facilitate collaboration with these key stakeholders in relation to dog welfare. Future research should focus on ensuring behaviour change theories are considered in strategy designed to address challenges, specifically in relation to educational campaigns and strategies focused on strengthening stakeholder relationships.

### Supplementary Information

**Additional file 1. **Supplementary material.

## Data Availability

The survey will be made available upon request  from the corresponding author.
