# Supplementary material for: A mixed-method survey to understand the role of dog welfare organisations in Ireland, including reported challenges and potential solutions
Source: Ir Vet J. 2023 Sep 30;76:27. doi: 10.1186/s13620-023-00249-6 (PMC10542677; doi:10.1186/s13620-023-00249-6)
Supplement: Supplementary file 1 — Additional file 1. Supplementary material. [file 13620_2023_249_MOESM1_ESM.docx]

*This document details the questions from the survey included within this study.*

**Questionnaire Items**

**Organisation full name _____________________________**

**Organisation abbreviation (if relevant) ____________________________**

**Please indicate in which county your organisation is situated
…………………………….**

**Please indicate the district, locality, or townland of your organisation
……………………………….**

**When was your organisation first** **registered with the Charities Regulatory Authority (CRA) in Ireland?**

*First registration date (month/year) _____________________________*

*What is your CRA number? _____________________________*

**Which of the following species did your organisation care for during 2021?**

*[TICK ALL THAT APPLY]*

| **Animal species** | **Tick all that apply** |
| --- | --- |
| *Dogs* |  |
| *Cats* |  |
| *Horses, donkeys or ponies* |  |
| *Cattle* |  |
| *Sheep* |  |
| *Goats* |  |
| *Pigs* |  |
| *Poultry or other birds* |  |
| *Exotics and small mammals (rabbits, guinea pigs, reptiles etc)* |  |
| *Wildlife species <please specify>* |  |

*Further detail of ‘Wildlife species’ __________________*

**During 2021, did your organisation use any fosterers to care for dogs?**

*[PLEASE CHOOSE ONLY ONE RESPONSE]*

a) Yes

b) No

**Does your organisation have a written policy on the following welfare actions: euthanasia, re-homing or assessment of potential owner suitability?**

*[PLEASE PROVIDE A YES OR NO ANSWER FOR EACH]*

| **Welfare action** | **Yes** | **No** |
| --- | --- | --- |
| *Euthanasia* |  |  |
| *Re-homing* |  |  |
| *Assessment of potential owner suitability* |  |  |

| **Welfare action** | **Yes** | **No** |
| --- | --- | --- |
| *Feeding Routines* |  |  |
| *Housing* |  |  |
| *Cleaning* |  |  |

**Does your organisation have a written policy or protocol on the following procedures?***[PLEASE PROVIDE A YES OR NO ANSWER FOR EACH]*

**Which of the following is used by your organisation to inform the public about dogs available for rehoming?**

*[TICK ALL THAT APPLY]*

| **Methods to inform public** | **Tick all that apply** |
| --- | --- |
| *Word of mouth* |  |
| *Social media (Facebook, Instagram, WhatsApp, forums etc.)* |  |
| *Newspapers* |  |
| *Other Websites (non-social media or non-newspaper)* |  |
| *E-mail (Circulation lists)* |  |
| *None of the above* |  |
| *Other <please specify>* |  |

*Further detail if ‘Other’ __________________*

**Which of the following procedures is conducted by your organisation prior to release of a dog to a new owner?**

*[TICK ALL THAT APPLY]*

| **Procedures conducted** | **Tick all that apply** |
| --- | --- |
| *A home visit* |  |
| *A request for proof of income to determine ability to provide proper care* |  |
| *A verification of prospective owner experience with dogs or other companion animals* |  |
| *A screening of prospective owner for previous breaches of animal welfare legislation* |  |
| *A check (or verification) on the number and type of animals already in the home* |  |
| *Organisation of trial visits (with prospective owners)* |  |
| *Other <please specify>* |  |

*Further detail if ‘Other’ __________________*

**When a dog is rehomed, what fees does your organisation charge to a new owner?**

*[PLEASE CHOOSE ALL THAT APPLY]*

|  | **Tick all that apply** |
| --- | --- |
| *No fees are charged* |  |
| *Administration fees* |  |
| *Cost of vaccination* |  |
| *Cost of neutering* |  |
| *Cost of microchipping* |  |
| *Cost of deworming* |  |
| *Cost of transportation* |  |
| *Cost of EU passports* |  |
| *A fixed amount (i.e., suggested donation)* |  |
| *Voluntary donation* |  |
| *Other <please specify>* |  |

*Further detail if ‘Other’ __________________*

**Does your organisation have a written record of all persons who adopted dogs from your organisation during 2021?**

*[PLEASE CHOOSE ONLY ONE RESPONSE]*

1. Yes
2. No

**When a dog is rehomed by your organisation, is there routine follow-up by your organisation to check on how the dog is being cared for?**

*[PLEASE CHOOSE ONLY ONE RESPONSE]*

1. Yes
2. No

**Please indicate the three most common reasons for euthanasia of dogs under your care in 2019 (prior to COVID-19) and in 2021?**

*[SEPARATELY FOR THESE TWO YEARS, PLEASE PLACE A “1”, “2”, AND “3”, WITH “1” BEING THE MOST COMMON REASON AND “3” BEING THE THIRD MOST COMMON REASON]*

|  | **2019**  ***(prior to COVID-19)*** | **2021** |
| --- | --- | --- |
| *Physical injury* |  |  |
| *Dog bite/aggression* |  |  |
| *Behavioural issue (non-aggression)* |  |  |
| *Injury to livestock or livestock worrying* |  |  |
| *Unable to rehome* |  |  |
| *Other 1 <please specify>* |  |  |
| *Other 2 <please specify>* |  |  |
| *Other 3 <please specify>* |  |  |

**During 2019 and 2021, please provide the number of dogs that left the care of your organisation annually for destinations within the Republic of Ireland for the following reasons.***[PLEASE PUT A NUMBER IN EACH BOX]*

|  | **Number of dogs** | |
| --- | --- | --- |
| **Possible destinations** | **2019** | **2021** |
| 1. *Reclaimed by original owner* |  |  |
| 1. *Rehomed to new private owner* |  |  |
| 1. *Transferred to other charity* |  |  |
| 1. *Transferred to a breeder* |  |  |
| 1. *Other 1 <please specify>* |  |  |
| 1. *Other 2 <please specify>* |  |  |
| 1. *Other 3 <please specify>* |  |  |

***Dogs leaving your organisation for Northern Ireland***

**Does your organisation send dogs to Northern Ireland?**

*[CHOOSE ONLY ONE RESPONSE]*

1. Yes
2. No

**If yes, why does your organisation send dogs to Northern Ireland?**

*[PLEASE TICK ALL THAT APPLY]*

| **Reasons for sending dogs to Northern Ireland** | **Tick all that apply** |
| --- | --- |
| 1. *Because there are insufficient eligible owners for rehoming dogs in the Republic of Ireland.* |  |
| 1. *Because there are more suitable owners for rehoming dogs in Northern Ireland.* |  |
| 1. *Because there are more suitable charities/organisations in Northern Ireland for rehoming dogs.* |  |
| 1. *Because financial contributions from rehoming of dogs are higher in Northern Ireland.* |  |
| 1. *Because of contractual agreements with funders or other agencies/charities.* |  |
| 1. *Other <please specify>* |  |

*Further detail if ‘Other’ __________________*

**During 2019 and 2021, please provide the number of dogs leaving the care of your organisation annually for the following reasons in Northern Ireland due to the following reasons.***[PLEASE PUT A NUMBER IN EACH BOX]*

|  | **Number of dogs** | |
| --- | --- | --- |
| **Possible destinations** | **2019** | **2021** |
| 1. *Rehomed to new private owner* |  |  |
| 1. *Transferred to other charity* |  |  |
| 1. *Transferred to a breeder* |  |  |
| 1. *Other 1 <please specify>* |  |  |
| 1. *Other 2 <please specify>* |  |  |
| 1. *Other 3 <please specify>* |  |  |

**Does your organisation send dogs to Great Britain?**

*[CHOOSE ONLY ONE RESPONSE]*

1. Yes
2. No

**If yes, why does your organisation send dogs to Great Britain?**

*[PLEASE TICK ALL THAT APPLY]*

| **Reasons for sending dogs to Great Britain** | **Tick all that apply** |
| --- | --- |
| 1. *Because there are insufficient eligible owners for rehoming dogs in the Republic of Ireland.* |  |
| 1. *Because there are more suitable owners for rehoming dogs in Great Britain.* |  |
| 1. *Because there are more suitable charities/organisations in Great Britain for rehoming dogs.* |  |
| 1. *Because financial contributions from rehoming of dogs are higher in Great Britain.* |  |
| 1. *Because of contractual agreements with funders or other agencies/charities.* |  |
| 1. *Other <please specify>* |  |

*Further detail if ‘Other’ __________________*

**During 2019 and 2021, please provide the number of dogs leaving the care of your organisation for destinations in Great Britain due to the following reasons.***[PLEASE PUT A NUMBER IN EACH BOX]*

|  | **Number of dogs** | |
| --- | --- | --- |
| **Possible destinations** | **2019** | **2021** |
| 1. *Rehomed to new private owner* |  |  |
| 1. *Transferred to other charity* |  |  |
| 1. *Transferred to a breeder* |  |  |
| 1. *Other 1 <please specify>* |  |  |
| 1. *Other 2 <please specify>* |  |  |
| 1. *Other 3 <please specify>* |  |  |

**Does your organisation send any dogs to countries other than the UK?**

*[CHOOSE ONLY ONE RESPONSE]*

1. Yes
2. No

**If yes, why does your organisation send dogs to countries other than the UK?**

*[PLEASE TICK ALL THAT APPLY]*

| **Reasons for sending dogs to countries other than the UK** | **Tick all that apply** |
| --- | --- |
| 1. *Because there are insufficient eligible owners for rehoming dogs in the Republic of Ireland.* |  |
| 1. *Because there are more suitable owners for rehoming dogs outside of the UK.* |  |
| 1. *Because there are more suitable charities/organisations outside of the UK for rehoming dogs.* |  |
| 1. *Because financial contributions from rehoming dogs are higher outside of the UK.* |  |
| 1. *Because of contractual agreements with funders or other agencies/charities.* |  |
| 1. *Other <please specify>* |  |

*Further detail if ‘Other’ __________________*

**The following** **questions pertain to the top three countries outside the Republic of Ireland and UK to which your organisation sent dogs during 2019 and 2021.**

1. **Firstly, please complete the following table with respect to the country (excluding the Republic of Ireland and UK) that received the largest number of dogs from your organisation during 2019 and 2021.**

**How many dogs were sent to this country each year over this period?***(Please put a number in each box. If data are not available for any of the boxes, please put "-" in these boxes to allow you to progress to the next question)*

| **Country 1** | **Number of dogs** | |
| --- | --- | --- |
| **Name** | **2019** | **2021** |
| *<Name of country>* |  |  |

**Over these five years,** **what types of establishments in this country did these dogs go to?**

*[PLEASE TICK ALL THAT APPLY]*

| **Country 1** | **Tick all that apply** |
| --- | --- |
| 1. *Private owner* |  |
| 1. *Other charity* |  |
| 1. *Breeder* |  |
| 1. *Other <please specify>* |  |

*If other, please provide details ____________________*

1. **Complete the following table with respect to the country (exclude the Republic of Ireland and UK) that received the second largest number of dogs from your organisation** **during 2019 and 2021.**

**How many dogs were sent to this country each year over this period?***(Please put a number in each box. If data are not available for any of the boxes, please put "-" in these boxes to allow you to progress to the next question)*

| **Country 2** | **Number of dogs** | |
| --- | --- | --- |
| **Name** | **2019** | **2021** |
| *<Name of country>* |  |  |

**Over these five years, what types of establishments in this country did these dogs go to?**

*[PLEASE TICK ALL THAT APPLY]*

| **Country 2** | **Tick all that apply** |
| --- | --- |
| *a) Private owner* |  |
| *b) Other charity* |  |
| *c) Breeder* |  |
| *d)Other <please specify>* |  |

*If other, please provide details ____________________*

1. **Complete the following table with respect to the country (exclude the Republic of Ireland and UK) that received the third largest number of dogs from your organisation during 2019 and 2021.**

**How many dogs were sent to this country each year over this period?***(Please put a number in each box. If data are not available for any of the boxes, please put "-" in these boxes to allow you to progress to the next question)*

| **Country 3** | **Number of dogs** | |
| --- | --- | --- |
| **Name** | **2019** | **2021** |
| *<Name of country>* |  |  |

**Over these five years, what types of establishments in this country did these dogs go to?**

*[PLEASE TICK ALL THAT APPLY]*

| **Country 3** | **Tick all that apply** |
| --- | --- |
| *a) Private owner* |  |
| *b) Other charity* |  |
| *c) Breeder* |  |
| *d)Other <please specify>* |  |

*If other, please provide details ____________________*

**Please indicate your agreement with the following statements.**

**I believe…**

|  | **Strongly disagree** | **Disagree** | **Neither agree nor disagree** | **Agree** | **Strongly Agree** |
| --- | --- | --- | --- | --- | --- |
| *…* *all animal welfare organisations in the Republic of Ireland should be registered with the Charities Regulatory Authority in Ireland?* |  |  |  |  |  |
| *…* *the Department of Agriculture, Food and the Marine should introduce minimum operational and animal welfare standards for all animal rescue organisations (including individuals who rescue animals)?* |  |  |  |  |  |

**Is your organisation a member of the Association of Dogs and Cats Homes (https://adch.org.uk)?***[PLEASE CHOOSE ONLY ONE RESPONSE]*a) Yes
b) No

**The following challenges typically affect an organisation’s ability to positively impact the welfare of dogs. For each challenge listed please** **indicate the extent in which you agree or disagree that these challenges apply to your organisation**

| **Main challenges** | **Strongly disagree** | **Disagree** | **Neither agree nor disagree** | **Agree** | **Strongly Agree** |
| --- | --- | --- | --- | --- | --- |
| 1. *Insufficient staff* |  |  |  |  |  |
| 1. *Insufficient staff with suitable training* |  |  |  |  |  |
| 1. *A general lack of funding* |  |  |  |  |  |
| 1. *Lack of resources to meet costs of re-homing abroad (including certification, transport, etc)* |  |  |  |  |  |
| 1. *Insufficient capacity to handle the number of dogs (supply and/or demand)* |  |  |  |  |  |
| 1. *Difficulties or an inability to re-homing particular dog breeds* |  |  |  |  |  |
| 1. *A lack of sufficient coordination with other dog welfare organisations* |  |  |  |  |  |
| 1. *Difficulties for organisation to comply with government requirements* |  |  |  |  |  |
| 1. *Insufficient engagement with local authorities* |  |  |  |  |  |
| 1. *A general lack of awareness among the public of the importance of dog welfare* |  |  |  |  |  |
| 1. *Other <please specify>* |  |  |  |  |  |

*If other, please provide details ____________________*

**How helpful do you think the following solutions would be to help you address the challenges your organisation experiences*.***

|  | **Not at all Helpful** | **Slightly Helpful** | **Moderately Helpful** | **Very Helpful** | **Extremely helpful** |
| --- | --- | --- | --- | --- | --- |
| Subsidised programmes (vaccination neutering & microchipping) |  |  |  |  |  |
| Access to standardised training for volunteers and employees |  |  |  |  |  |
| Access to resources to educate potential owners (i.e., breed suitability) |  |  |  |  |  |
| Rigorous enforcement of recommendations/ policies |  |  |  |  |  |
| Opportunity to attend conferences or seminars with other welfare organisations |  |  |  |  |  |
| Greater clarity on the criteria for government financial grants |  |  |  |  |  |
| Other |  |  |  |  |  |

*If other, please provide details ____________________*

**What solutions do you feel would be the most effective in addressing the challenges you face? And Why?**

*[PLEASE PARTICULARLY CONSIDER THE CHALLENGES THAT YOU PRIORITISED IN THE PREVIOUS QUESTION]*

**At this point in time, what does your organisation see as the broader challenges to dog welfare in the Republic of Ireland (ie excluding Northern Ireland)?**

*[PLEASE RANK THE TOP 5 CHALLENGES IN ORDER OF IMPORTANCE (1 BEING THE MOST IMPORTANT CHALLENGE)]*

| **Main challenges** | **Indicate 1 to 5** |
| --- | --- |
| 1. *A general lack of awareness among the public of the importance of dog welfare* |  |
| 1. *Irresponsible pet ownership (such as a lack of owner education during the initial selection of dogs, owners obtaining dogs without long-term planning)* |  |
| 1. *A preference among the public for ‘fashionable’ or popular dog breeds* |  |
| 1. *Insufficient opportunities for rehoming of dogs in the Republic of Ireland* |  |
| 1. *A general lack of compliance by the public with existing government requirements (licensing, microchipping, etc)* |  |
| 1. *Inadequate national legislation (including a need to update existing legislation, given current challenges)* |  |
| 1. *A lack of regulation of the dog welfare organisations (for example, too many unregistered charities)* |  |
| 1. *Insufficient funding from government* |  |
| 1. *Other <please specify>* |  |

*If other, please provide details ____________________*

**What does your organisation see as solutions to these broad challenges to dog welfare in the Republic of Ireland?***[PLEASE PARTICULARLY CONSIDER THE 5 CHALLENGES THAT YOU PRIORITISED IN THE PREVIOUS QUESTION]*

**Finally, please add any additional comments you have**

**THANK YOU**
